# The Effect of Alendronate on Osteoclastogenesis in Different Combinations of M-CSF and RANKL Growth Factors

**DOI:** 10.3390/biom11030438

**Published:** 2021-03-16

**Authors:** Věra Hedvičáková, Radmila Žižková, Matěj Buzgo, Michala Rampichová, Eva Filová

**Affiliations:** 1 Department of Tissue Engineering, Institute of Experimental Medicine, The Czech Academy of Sciences, Videnska 1083, 142 20 Prague, Czech Republic; radmila.zizkova@iem.cas.cz (R.Ž.); matej@inocure.cz (M.B.); michala.rampichova@iem.cas.cz (M.R.); eva.filova@iem.cas.cz (E.F.); 2 Department of Chemistry, Faculty of Science, Humanities and Education, Technical University of Liberec, Studentska 1402/2, 461 17 Liberec, Czech Republic; 3 InoCure, Politických Vězňů 935/13, 110 00 Praha, Czech Republic

**Keywords:** alendronate, M-CSF, osteoclastogenesis, RANKL

## Abstract

Bisphosphonates (BPs) are compounds resembling the pyrophosphate structure. BPs bind the mineral component of bones. During the bone resorption by osteoclasts, nitrogen-containing BPs are released and internalized, causing an inhibition of the mevalonate pathway. As a consequence, osteoclasts are unable to execute their function. Alendronate (ALN) is a bisphosphonate used to treat osteoporosis. Its administration could be associated with adverse effects. The purpose of this study is to evaluate four different ALN concentrations, ranging from 10^−6^ to 10^−10^ M, in the presence of different combinations of M-CSF and RANKL, to find out the effect of low ALN concentrations on osteoclastogenesis using rat and human peripheral blood mononuclear cells. The cytotoxic effect of ALN was evaluated based on metabolic activity and DNA concentration measurement. The alteration in osteoclastogenesis was assessed by the activity of carbonic anhydrase II (CA II), tartrate-resistant acid phosphatase staining, and actin ring formation. The ALN concentration of 10^−6^ M was cytotoxic. Low ALN concentrations of 10^−8^ and 10^−10^ M promoted proliferation, osteoclast-like cell formation, and CA II activity. The results indicated the induction of osteoclastogenesis with low ALN concentrations. However, when high doses of ALN were administered, their cytotoxic effect was demonstrated.

## 1. Introduction

Bone is a rigid connective tissue that enables locomotion, supports weight, protects the internal organs, and maintains mineral homeostasis. As it is a dynamic type of tissue, the process of continuous bone remodeling occurs throughout life. The bones consist of several cell types that are orchestrated together in order to balance the process of bone formation, executed by osteoblasts and bone resorption carried out by osteoclasts. The balance is maintained by the set of growth factors (GFs) and cytokines. The most important include macrophage-colony stimulating factor (M-CSF) and receptor activator of nuclear factor kappa B ligand (RANKL), which bind to the cell surface receptors of osteoclast precursors [1]. The balance of signaling is negatively influenced by a soluble glycoprotein receptor called osteoprotegerin (OPG). OPG binds to RANKL, thereby preventing excessive bone resorption [2].

An imbalance between these molecular mechanisms results in a pathophysiological change in bones, resulting in impaired bone strength and a high risk of fracture, mainly in elderly or osteoporotic people. The subsequent healing period is often prolonged, which has an impact on national healthcare [3,4]. The first choice treatment for osteoporosis is bisphosphonates (BPs). Bisphosphonates resemble pyrophosphates, giving them the ability to bind to bone minerals [5]. Upon binding to bone minerals, BPs can be released by the action of osteoclasts and internalized [6]. There are two classes of BPs based on the R2 substituents. Nitrogen-containing BPs (N-BPs) contain nitrogen in R2. N-BPs are, for example, alendronate (ALN) and risendronate. Non-nitrogen-containing BPs such as clodronate or etidronate are contained in R2 Cl or CH_3_, respectively. Diverse R2 side chains confer in different modes of action [7]. N-BPs inhibit enzymes in the mevalonate pathway, which subsequently affect the prenylation of proteins, e.g., small GTPases [8]. The exact mode of action is believed to be complex but remains unknown. An impaired ruffled border, important for the osteoclasts to execute bone-resorbing activities, is one of the outcomes [7,9]. Non-nitrogen-containing BPs are metabolized into a toxic analog of ATP that is nonhydrolyzable [10].

Nowadays, there is growing evidence of in vivo side-effects caused by long-term exposure to ALN treatment, such as reduced bone strength, atypical fractures, or bisphosphonate-associated osteonecrosis of the jaw [11,12]. In order to improve the patient’s prospects for quality movement, accelerated fracture healing by local treatment is being investigated [13]. Toker et al. demonstrated no difference between fracture healing after local or systemic delivery of ALN [14]. Moreover, local delivery requires lower doses of ALN while decreasing the negative impact of its systemic administration. Drug delivery is an elegant solution for the healing of defects, where the action of drugs or other active molecules is necessary. Active molecules can be incorporated directly inside the scaffolds [15,16] or into microparticles, which can subsequently be immobilized on the scaffold surface [17]. Thus, scaffolds can be used as cell-free implants for bone tissue engineering. The efficient concentrations of the molecules have to be set, and their effect on suitable cell types has to be evaluated. The drug delivery system has to be balanced to release the effective concentrations for the time period necessary for healing.

The purpose of this study is to find out the efficient concentration of ALN for the inhibition of osteoclast formation and activity for local drug delivery. The concentration range of ALN was set between 10^−6^ and 10^−10^ M. The highest used concentration of ALN was set to 10^−6^ M, with the aim of serving as a negative control because this concentration has been found to be cytotoxic for other cell types [18,19,20]. ALN was chosen as a widely used representative of N-BPs. Peripheral blood mononuclear cells (PBMCs) were seeded on tissue-cultured polystyrene as ALN can also be internalized by cultured cells from the media, demonstrated by Coxon et al. [21]. Moreover, as low concentrations of ALN positively influence interleukin 6 (IL-6) production [22], the effect on osteoclast-like cell formation from PBMCs was evaluated at the same time. In vitro, the induction of osteoclastogenesis in the monoculture of PBMCs is stimulated by adding M-CSF and RANKL into the culture media [23]. In osteoporotic conditions, the signaling is altered; therefore, PBMCs were cultured in the presence or absence of both M-CSF and RANKL or with only one of the mentioned GFs. As the osteoporotic model is often studied on small rodents [4,13,24], the effect of ALN was evaluated on primary PBMCs isolated from rat whole-blood. However, the effect of ALN can vary between different species; therefore, we have investigated the effect of ALN on human PBMCs as well.

## 2. Materials and Methods

### 2.1. Cell Isolation and Seeding

hPBMCs were isolated from whole human blood. All donors formally consented to the use of their blood for scientific purposes. The study was conducted in accordance with the Institute of Experimental Medicine CAS, and the protocol was approved by the Ethics Committee under File No. 2020/03.

rPBMCs were isolated from whole-blood aspirated from six-month-old Wistar rats by cardiac puncture under the inhalation of isoflurane. The Ethical Principles and Guidelines for Scientific Experiments on Animals were respected throughout the study. The maintenance and handling of the experimental animals followed EU Council Directive 86/609 EEC, and the animals were treated in accordance with the principles of Care and Use of Animals.

The peripheral blood with anticoagulants was diluted in Hanks’ balanced salt solution (HBSS) at a 1:1 ratio. The suspension was layered over Ficoll–Paque and centrifuged at 1410 rpm (NF800R centrifuge, Nüve, Ankara, Turkey) in a swing-out rotor for 30 min. The mononuclear cell layer was aspirated, transferred into a conical tube, mixed with HBSS, and centrifuged at 1500 rpm for 10 min at 16 °C. The supernatant was removed, and the cells were resuspended in HBSS and centrifuged at 1250 rpm for 10 min at 16 °C. The supernatant was removed and cells were resuspended in HBSS and centrifuged at 800 rpm for 10 min at 16 °C. The supernatant was removed, and cells were mixed with Dulbecco’s modified Eagle’s medium, 2 mM L-glutamine, 10% fetal bovine serum, and 1% penicillin/streptomycin. rPBMCs were pooled. On Day 0, PBMCs were counted and seeded in a 96-well plate, 1 × 10^5^ per well. After 12 h, the media was changed; for specific groups, diverse concentrations of alendronate sodium (Sigma Aldrich, ShangHai, China) and 25 ng mL^−1^ M-CSF (Peprotech, London, UK) were added. After 24 h, the media was changed. For specific groups, 30 ng mL^−1^ RANKL (Peprotech, London, UK) was added. Sample naming and culture conditions are stated in Table 1. The media was changed twice a week.

### 2.2. Metabolic Activity and DNA Quantification Measurement

On Days 6 and 15, the metabolic activity of cells was measured using an MTS assay (CellTiter 96^®^ AQueous One Solution Cell Proliferation Assay; Promega, Madison, WI, USA), according to the manufacturer’s manual. Lysis buffer was further added to the samples, and the amount of DNA was measured using the Quant-iT™ dsDNA Assay Kit (Life Technologies, Eugene, OR, USA) according to the manufacturer’s manual. The data were processed using the calibration curve of the standards from the kit.

### 2.3. Carbonic Anhydrase II Activity

The activity of carbonic anhydrase II (CA II) was evaluated on Days 6 and 15, according to the methods used elsewhere [12]. Briefly, PBMCs were lysed in 100 µL of 1% Triton X-100 in phosphate buffer saline (PBS) on ice for 50 min, followed by 10 min of shaking. Then, 50 µL of cell lysate was mixed with 50 µL of reaction buffer (12.5 mM TRIS, 75 mM NaCl, 2 mM 4-nitrophenyl acetate; pH = 7.5) and measured after 1 h at 405 nm (Tecan Infinite M200, Männedorf, Switzerland). Standards of 4-nitrophenol, in the range of 0.125–2 mM, were used for the calibration curve.

### 2.4. Tartrate-Resistant Acid Phosphatase and Hematoxylin Staining

Tartrate-resistant acid phosphatase (TRAP) staining was performed using a TRAP Kit (Sigma Aldrich, St. Louis, MO, USA) according to the manufacturer’s manual. A hematoxylin solution was then used to stain the nuclei. The cells were visualized by light microscopy (Olympus U-RFL-T, IX-51, Olympus, Tokyo, Japan).

### 2.5. Actin Cytoskeleton Staining

The formation of the actin ring was visualized on Days 6 and 15. The cells were fixed with 2% paraformaldehyde in PBS for 20 min. Thereafter, 0.5% Triton-X-100 was incubated with the cells for 2 min, with subsequent thorough PBS washing. Phalloidin-Atto 633 (10 nM, Sigma Aldrich, Steinheim, Germany) 1:500 was added for 45 min. Cell nuclei were visualized using Hoechst 34580 (10 ng mL^−1^, Invitrogen, Carlsbad, CA, USA), diluted 1:1500 for 15 min. The cells were visualized using an epifluorescence microscope (Olympus U-RFL-T, IX-51, Olympus, Tokyo, Japan).

### 2.6. Statistical Analysis

Quantitative data are presented as mean values ± standard deviation (SD). The averaged values were determined from at least 5 independently prepared samples. The results were evaluated statistically using SigmaStat 12.0 (Systat, San Jose, CA, USA). If the data passed the normality test and the test of equality of variances, statistical significance between a pair of groups was determined by ANOVA test and Tukey’s comparative test for posthoc analysis. If the data were without normal distribution, statistical significance between a pair of groups was determined using the Kruskal-Wallis one-way analysis of variance on ranks and Dunn’s multiple comparisons test for posthoc analysis. All results were considered statistically significant if *p* was < 0.05.

## 3. Results

In this study, the rat and human PBMCs were cultured in different culture conditions. Diverse concentrations of ALN in either the presence or absence of 25 ng mL^−1^ M-CSF and 30 ng mL^−1^ RANKL were used. See Table 1 for specific culture conditions and sample names.

**Table 1:** Sample name list of the experimental groups and the used concentrations of alendronate (ALN), macrophage-colony stimulating factor (M-CSF), and receptor activator of nuclear factor kappa B ligand (RANKL).

Sample Name	ALN [M]	M-CSF [ng mL^−1^]	RANKL [ng mL^−1^]	Sample Name	ALN [M]	M-CSF [ng mL^−1^]	RANKL [ng mL^−1^]
A10^−6^+MR	10^−6^	25	30	A10^−8^+M	10^−8^	25	-
A10^−6^	10^−6^	-	-	A10^−8^+R	10^−8^	-	30
A10^−6^+M	10^−6^	25	-	A10^−10^+MR	10^−10^	25	30
A10^−6^+R	10^−6^	-	30	A10^−10^	10^−10^	-	-
A10^−7^+MR	10^−7^	25	30	A10^−10^+M	10^−10^	25	-
A10^−7^	10^−7^	-	-	A10^−10^+R	10^−10^	-	30
A10^−7^+M	10^−7^	25	-	A0+MR	-	25	30
A10^−7^+R	10^−7^	-	30	A0	-	-	-
A10^−8^+MR	10^−8^	25	30	A0+M	-	25	-
A10^−8^	10^−8^	-	-	A0+R	-	-	30

### 3.1. DNA Quantification

**Figure 1:**
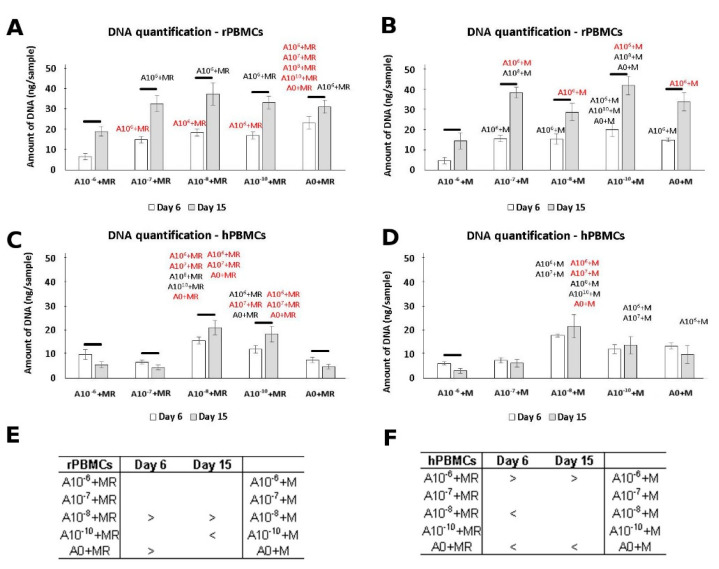
Peripheral blood mononuclear cells (PBMCs), with proliferation measured using the Quant-iT™ dsDNA Assay Kit to quantify the amount of DNA. rPBMCs cultured in the presence of M-CSF and RANKL (**A**); rPBMCs cultured in the presence of M-CSF (**B**); hPBMCs cultured in the presence of M-CSF and RANKL (**C**); hPBMCs cultured in the presence of M-CSF (**D**). Statistical significance is denoted above the columns. Statistical significance among the tested groups within one experimental day is given by sample name (black *p* < 0.05, red *p* < 0.001). Comparison within the same group between Days 6 and 15 is marked with a line above the respective columns. Comparison across the groups with the same concentration of ALN on Days 6 and 15 was performed to identify the pair-wise differences. It is stated in Tables (**E**) and (**F**) for rat and human PBMCs, respectively (*p* < 0.05). Data are presented as mean ± standard deviation.

The amount of DNA was quantified as an indicator of cell proliferation. Based on the obtained data, the lowest amount of DNA was detected in the presence of 10^−6^ M of ALN on both tested days (Figure 1A,B). A higher rate of PBMCs proliferation was detected in groups A10^−8^+M and A0+MR on Day 6. A statistically significant increase in the amount of DNA was detected between Days 6 and 15; the amount of DNA reached comparable values. However, a higher amount of DNA remained in the A10^−8^+M group. Clearly, while hPBMCs were cultured in diverse conditions, the concentrations of 10^−8^ and 10^−10^ M of ALN promoted the highest proliferation compared to all other tested concentrations (Figure 1E,F). On the other hand, an increase in the amount of DNA between Days 6 and 15 was only detected in the A10^−8^+MR and A10^−10^+MR groups.

Evidently, ALN in the concentration of 10^−6^ negatively affected PBMCs proliferation. On the other hand, lower concentrations of ALN resulted in an increase in DNA quantification for both rat and human PBMCs. Culture conditions with a media without GFs or containing only RANKL affected the adhesion of the PBMCs. Only a low amount of DNA was detected in the groups without M-CSF in the media (Figure S1). Moreover, the amount of DNA decreased up to Day 15. Therefore, further data for these culture conditions are not shown.

### 3.2. Metabolic Activity

**Figure 2:**
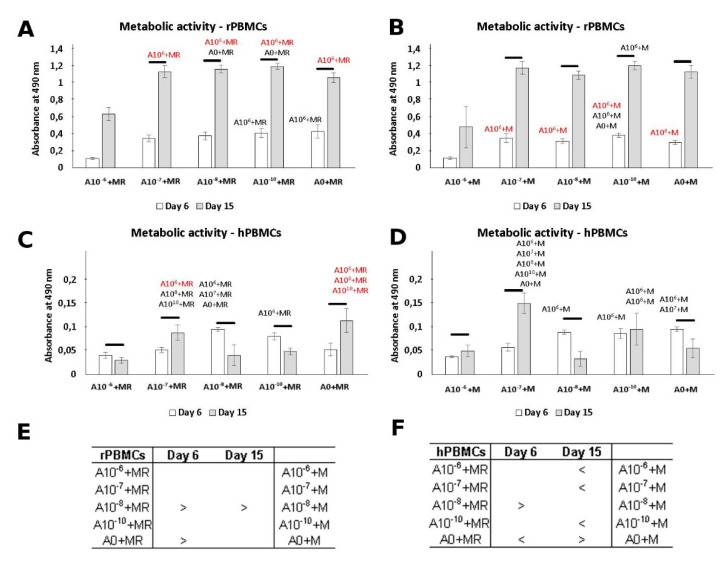
Metabolic activity of peripheral blood mononuclear cells (PBMCs) measured by MTS assay. rPBMCs cultured in the presence of M-CSF and RANKL (**A**); rPBMCs cultured in the presence of M-CSF (**B**); hPBMCs cultured in the presence of M-CSF and RANKL (**C**); hPBMCs cultured in the presence of M-CSF (**D**). Statistical significance is denoted above the columns. Statistical significance among the tested groups within one experimental day is given by sample name (black *p* < 0.05, red *p* < 0.001). Comparison within the same group between Days 6 and 15 is marked with a line above the respective columns. Comparison across the groups, with the same concentration of ALN on Days 6 and 15, was performed to identify the pair-wise differences. It is stated in Tables (**E**) and (**F**) for rat and human PBMCs, respectively (*p* < 0.05). Data are presented as mean ± standard deviation.

The cytotoxicity of ALN was measured on Days 6 and 15 (Figure 2). The lowest metabolic activity was detected in groups cultured in the presence of 10^−6^ M ALN. This is in accordance with DNA quantification, as a lower amount of cells was detected for this culture condition. Increased metabolic activity was detected on Day 15, with comparable values across the groups treated with ALN and the control groups. A different trend in the metabolic activity of hPBMCs was observed (Figure 2). Although the culture condition with the highest ALN concentration remained with the statistically lowest metabolic activity, the overall increase in absorbance remained low.

In general, we could observe a trend in cytotoxicity when ALN, in the concentration of 10^−6^ M, was added into the culture media. However, by lowering the ALN concentration, the negative effect was dismissed. A positive increase in metabolic activity of PBMCs was detected even as the concentration of ALN was decreasing.

### 3.3. CA II Activity

**Figure 3:**
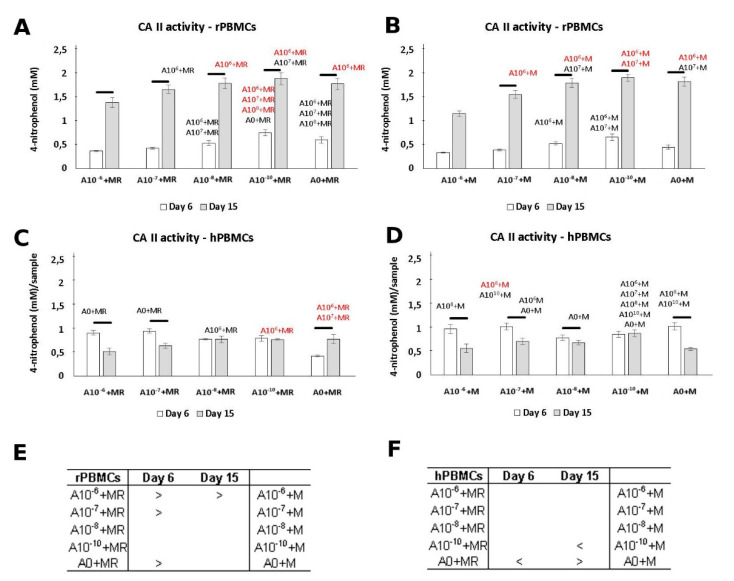
Carbonic anhydrase II (CA II) activity of peripheral blood mononuclear cells (PBMCs) quantified based on the concentration of formed 4-nitrophenol. rPBMCs cultured in the presence of M-CSF and RANKL (**A**); rPBMCs cultured in the presence of M-CSF (**B**); hPBMCs cultured in the presence of M-CSF and RANKL (**C**); hPBMCs cultured in the presence of M-CSF (**D**). Statistical significance is denoted above the columns. Statistical significance among the tested groups within one experimental day is given by sample name (black *p* < 0.05, red *p* < 0.001). Comparison within the same group between Days 6 and 15 is marked with a line above the respective columns. Comparison across the groups with the same concentration of ALN on Days 6 and 15 was performed to identify the pair-wise differences. It is stated in Tables (**E**) and (**F**) for rat and human PBMCs, respectively (*p* < 0.05). Data are presented as mean ± standard deviation.

The positive effect of ALN in a lower concentration was demonstrated by CA II activity measurement (Figure 3). CA II is an enzyme typical in osteoclasts [12]. rPBMCs showed higher CA II activity in the A10^−10^+MR and A10^−10^+M groups. A statistical increase in CA II activity was detected between Day 6 and Day 15. Additionally, statistical analysis across the groups revealed higher CA II activity, preferably in the groups treated with both GFs, M-CSF and RANKL (Figure 3C). A decreasing concentration of ALN in the culture media of rPBMCs increased the positive effect on CA II activity.

While the activity of CA II was increasing with the culture time in the case of rPBMCs, the trend was distinct in the case of hPBMCs. There was no observed increase, but even a slight decrease in the activity of CA II in the A10^−6^+MR, A10^−6^+M, and A0+M groups from Day 6 to Day 15.

### 3.4. Visualization of Formed Osteoclasts by TRAP and Hematoxylin Staining

**Figure 4:**
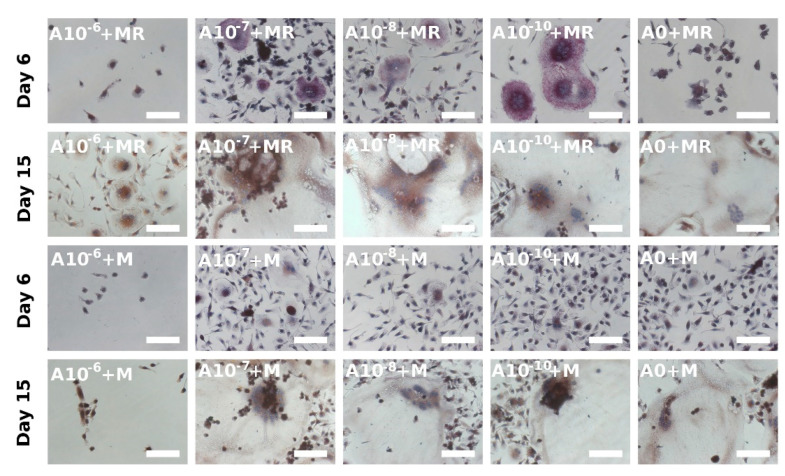
Osteoclast-like cell generation from rat peripheral blood mononuclear cells on Days 6 and 15 using a tartrate-resistant acid phosphatase-staining kit. Each image is labeled in the left upper corner by the name of the tested group. The day of visualization is indicated at the beginning of each row. Fused osteoclast-like cells were observed on Day 6 in groups with lower concentrations of ALN (A10^−7^+MR, A10^−8^+MR, A10^−10^+MR); however, with proceeding culture time, on Day 15, the fused cells were visible even in the A0+MR group. However, the area of fused cells was lower compared to the other tested groups. Culture conditions without RANKL in the culture media resulted in the fusion of the cells at later time points of the experiment. The positive trend of lower ALN concentration was observed again (A10^−7^+M, A10^−8^+M, A10^−10^+M groups). Scale bar 100 µm, magnification 200×.

TRAP is another typical osteoclastic enzyme [12]. During staining, hematoxylin was used to stain the nuclei; therefore, the osteoclasts, characterized as multinucleated cells with at least 3 nuclei, could be detected. It was noted that when the TRAP staining was visualized on Day 6, the osteoclast-like cell formation was clearly visible in the A10^−7^+M, A10^−8^+MR, and A10^−10^+MR groups (Figure 4), but in the A0+MR group, only the beginning of the osteoclastogenesis was observed. On the other hand, when rPBMCs were only cultured with M-CSF, the A10^−7^+M, A10^−8^+M, and A10^−10^+M groups were comparable with the control group, A0+M, where osteoclastogenesis was barely visible. In the A10^−6^+M and A10^−6^+M groups, with the highest concentration of ALN, only a few rounded rPBMCs were visualized. In summary, the negative effect of ALN, at the concentration of 10^−6^ M, was observed. Moreover, the lower concentrations of ALN, in fact, supported the process of osteoclast formation. As the culture time proceeded until Day 15, the constant adding of GFs in vitro resulted in the formation of osteoclast-like cells in each of the groups, with the exception of the A0+M group. However, the initial effect of low ALN concentration demonstrated an earlier induction of osteoclast-like cell formation from rPBMCs on Day 6 compared to the control groups, A0+MR and A0+M.

**Figure 5:**
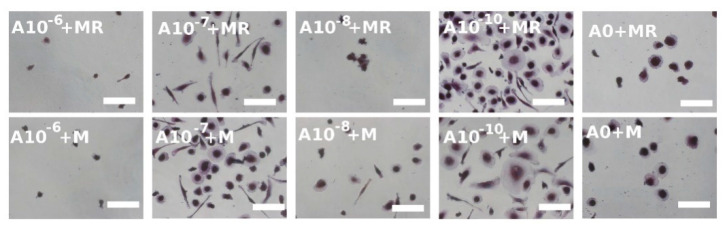
A tartrate-resistant acid phosphatase staining kit was used to stain human peripheral blood mononuclear cells on Day 15. The mononuclear cells were observed even with the proceeding culture period. However, in the A10^−10^+MR and A10^−10^+M groups, the beginning of fusion was observed. Scale bar 100 µm, magnification 200×.

Visualization of hPBMCs after the TRAP staining on Day 15 revealed that cells started to fuse in a less exemplary way compared to rPBMCs (Figure 5). No huge differences were observed when the two culture conditions, with both GFs or solely with M-CSF, were compared. The most spread and fused cells were formed in the groups with the lowest concentration of ALN, namely, the A10^−10^+MR and A10^−10^+M groups. Visualization of the hPBMCs, cultured in the media without GFs or only in the presence of RANKL, detected only a few adhered hPBMCs that were of rounded shape and not fused nor spread (see Figure S1).

### 3.5. Actin Ring Formation Staining

**Figure 6:**
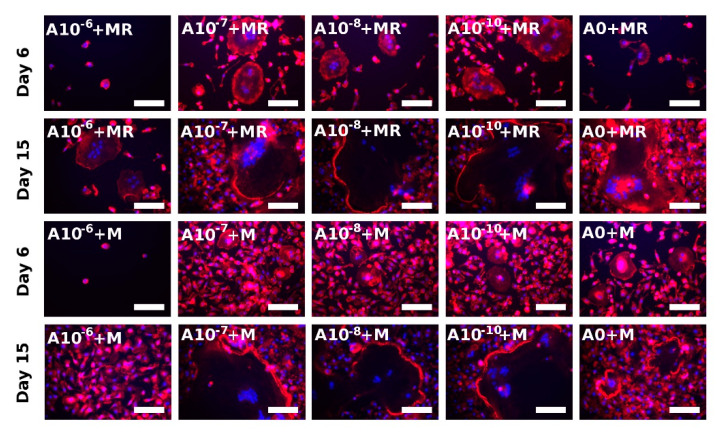
Visualization of actin ring formation of fused rat peripheral blood mononuclear cells on Days 6 and 15 using epifluorescence microscopy. Filamentous actin was stained with phalloidin (red colour), and nuclei were stained with Hoechst 34580 (blue color). Each image is labeled in the left upper corner by the name of the tested group. The day of visualization is indicated at the beginning of each row. In the presence of both M-CSF and RANKL, the fused rPBMCs were visualized on Day 6 in the A10^−7^+MR, A10^−8^+MR, and A10^−10^+MR groups, indicating the positive effect of ALN in lower concentrations. In the presence of only M-CSF, the fusion of cells began on later experimental days, and, on Day 15, fused cells were observed in A10^−7^+M, A10^−8^+M, and A10^−10^+M groups. Scale bar 100 µm, magnification 200×.

Actin ring formation is one of the indicators of the successful formation of active osteoclasts in vitro [23]. The visualization was performed on both Days 6 and 15 (Figure 6). The observation of rPBMCs was in agreement with the performed TRAP-staining method (Figure 4). The fusion of rPBMCs was preferably visible in the A10^−7^+MR, A10^−8^+MR, and A10^−10^+MR groups, but actin ring formation was not visible yet. However, with the proceeding culture time, the strongly stained actin ring became visible in the A0+MR, A10^−7^+MR, A10^−8^+MR, and A10^−10^+MR groups on Day 15. On Day 15, fused rPBMCs were observed even in the A10−^6^+MR group, even though no fused cells were detected in this group on Day 6. The state of formed osteoclast-like cells in the A10^−6^+MR group on Day 15 was comparable to Day 6 of the A10^−7^+MR, A10^−8^+MR, and A10^−10^+MR groups. In the A0+M, A10^−7^+M, A10^−8^+M, and A10^−10^+M groups, the beginning of actin ring formation was detected on Day 6. On Day 15, we observed an already well-formed and strongly stained actin ring. In the A10^−6^+M group, we observed only adhered single nuclear rPBMCs spread on the surface for the whole period of time.

The visualization of actin ring formation of hPBMCs was performed on Day 15. A strongly stained actin cytoskeleton was observed preferentially around the nuclei in all the groups, with the exception of the A10^−10^+MR and A10^−10^+M groups, where a formed acting ring was observed (Figure S2).

## 4. Discussion

In this study, the effect of ALN on initial osteoclastogenesis in a culture of rPBMCS and hPBMCs was examined. We expected to find an efficient concentration of ALN in a range of 10^−6^ and 10^−10^ M of ALN that, although not cytotoxic, would, however, result in the inhibition of osteoclast-like cell formation. The highest tested concentration (10^−6^ M) was found to be cytotoxic. On the other hand, lower ALN concentrations, 10^−8^ M and 10^−10^ M, were found to be supportive of metabolic activity, proliferation, and the induction of osteoclastogenesis in rat and human PBMCs.

ALN belongs to the group of N-BPs that are used to treat osteoporosis [25]. BPs are analogs of pyrophosphates, but, unlike pyrophosphates, the link between the two phosphate groups in ALN is created by C instead of O. BPs are then made resistant to hydrolysis; therefore, oral administration is possible [26]. However, resistance to hydrolysis may evince a negative influence on the mineralization of bone extracellular matrix by osteoblasts at higher doses of ALN [27,28]. The concentration known to possess a cytotoxic effect on different cultured cells is in the range of 10^−6^ to 10^−5^ M [18,19,20]. Therefore, the chosen culture conditions started with the highest concentration of ALN set to 10^−6^ M. The verification of cytotoxicity and the induction of osteoclastogenesis were performed using primary cells, rat and human PBMCs, rather than cell lines. In cell lines, the cell genotype is manipulated and, therefore, the behavior of cells can be altered. The follow-up of the ALN effect on the induction of osteoclastogenesis was performed long-term for 15 days of cultivation. To observe the effect during that period of time, two time points were set, Days 6 and 15.

The mechanism of the ALN effect in vivo is based on the ability of ALN to bind to Ca^2+^ within the bone mineral phase. The acidification of the environment in the resorption lacunae by osteoclast activity is followed by ALN internalization [6]. However, for ALN uptake by cells, it is not necessary to culture the cells on hydroxyapatite-like surfaces. Coxon et al. demonstrated that ALN was internalized by rabbit osteoclasts from the culture media, seeded on glass coverslips, [21]. Therefore, in this study, PBMCs were seeded on tissue-cultured polystyrene (TCP) as these cells are highly endocytic and, consequently, susceptible to ALN internalization from the medium.

The PBMCs were cultured in the presence or absence of M-CSF and RANKL growth factors. The concentration of M-CSF and RANKL used routinely in in-vitro conditions varies. In the study of Bernhardt et al., 25 ng mL^−1^ M-CSF and 50 ng mL^−1^ RANKL were used [29]. Kleinhans et al. used the concentration of 50 ng mL^−1^ for each of these GFs [30]. On the other hand, Penolazzi et al. used lower concentrations of both factors, 25 ng mL^−1^ M-CSF and 30 ng mL^−1^ RANKL [31]. The culture conditions of our experiment were set to 25 ng mL^−1^ of M-CSF and 30 ng mL^−1^ of RANKL, as higher doses of RANKL negatively regulate cell proliferation [32]. The culture conditions without M-CSF did not favor the adhesion of PBMCs; the cells remained rounded, with a single nucleus. On the other hand, in the presence of M-CSF, the PBMCs underwent the process of adhesion and fusion, typical for the induction of osteoclastogenesis. The observation confirmed the key role of M-CSF in cell adhesion [33,34].

The proliferation of rPBMCs cultured in the media with M-CSF was negatively influenced by ALN at the concentration of 10^−6^ M. The other tested concentrations of ALN did not show any effect on rPBMCS in the presence of both GFs in the culture media. However, when rPBMCs were cultured only with M-CSF, we observed a positive effect on cell proliferation in the culture with 10^−10^ M of ALN. Moreover, the positive effect of 10^−8^ M ALN and, to a lesser extent, of 10^−10^ M ALN was observed in the culture of hPBMCs. Metabolic activity measurement revealed a similar trend to DNA quantification analysis. During the cultivation of rPBMCs, the lowest metabolic activity was detected when 10^−6^ M of ALN was added to the culture media. However, the lowest concentration of ALN, 10^−10^ M, promoted an improvement of metabolic activity in rPBMCs.

The effect of ALN on the cell proliferation of osteoclasts and osteoclast precursors differs across the published studies. A positive effect of ALN at the concentration of 10^−5^ and 10^−7^ M was demonstrated by the superior proliferation of human THP-1-derived osteoclasts [35]. The negative effect of higher concentrations of ALN was proven in several studies. In contrast, Manzano-Moreno et al. observed increased apoptosis of murine osteoclasts treated with 5 × 10^−7^ M ALN [36]; 5 × 10^−5^ M ALN decreased the amount of DNA in mouse bone marrow-derived macrophages [37]; 10^−4^ M ALN negatively affected RAW cell proliferation. Furthermore, metabolic activity measurement revealed the negative effect of 10^−3^ M [19].

The effect on other cell types was also examined. Martins et al. detected a negative effect on cell proliferation of Saos2 cells at the concentration of 10^−5^ M ALN; 10^−6^ M was found to have no effect [19]. With 10^−5^ M ALN or higher, a decreased number of MG-63 cells was found [35,38]. Correia et al. found the concentration of 10^−6^ M ALN to inhibit the proliferation of human periodontal ligament fibroblasts. A lower concentration of ALN allowed cells to reach a confluent state [20]. Sung et al. observed decreased proliferation and metabolic activity of human rotator cuff tendon fibroblasts cultured in the presence of 10^−4^ M ALN. Lower concentrations were found to have no side effects [39]. ALN, in a concentration higher than 3 × 10^−6^ M, showed a cytotoxic effect and increased apoptosis in human dental pulp stem cells [40]. The concentrations negatively affecting cultured cells differ according to the cell type. Highly endocytic cell types are most likely to respond negatively to lower ALN concentrations compared to less endocytic cell types. Mathov et al. detected a positive effect of other N-BPs, olpadronate and pamidronate, on the proliferation of osteoblasts. The positive-acting mechanism of these N-BPs was due to the action of open calcium channels that led to the activation of ERK kinase, which positively influences the cell cycle [41].

The activity of the CA II enzyme supported the observed results of the cytotoxic effect of 10^−6^ M ALN. The CA II activity measured at this concentration was the lowest from all groups when rPBMCs were incubated in the presence of M-CSF. There is also a visible trend of CA II activity, which has a dependence on ALN concentration. The positive effect of the lowest ALN concentration, the A10^−10^+M group, on CA II activity was observed. On the contrary, the cultivation of hPBMCs did not reveal such a defined cytotoxic effect of the highest ALN concentration. On Day 15, we observed a concentration-dependence effect on CA II activity measurement when both GFs were added to the culture media. However, the culture conditions in the presence of only M-CSF on Day 15 revealed a positive effect of the lower ALN concentrations, 10^−10^ to 10^−7^ M, even in comparison to the control group without ALN.

TRAP staining and actin ring formation observation clearly confirmed the positive effect of low ALN concentrations, 10^−7^ to 10^−10^ M ALN, on the induction of osteoclastogenesis. It intensified the differences between the low ALN concentrations used and the control group in the early days of the experiment. The actin cytoskeleton staining method and the fusion of rPBMCs showed the initiation of osteoclast-like cell formation on Day 6. On Day 15, we detected a strongly stained actin ring and fused cells in all the groups, with the exception of the groups treated with 10^−6^ M ALN. The absence of RANKL in the culture media slowed the formation of osteoclast-like cells.

The effect of ALN on CA II, TRAP staining, and actin ring formation differs in the published studies. Zhang et al. reported decreasing mRNA expression and protein levels of CA II and TRAP in RAW cells cultured in the presence of 10^−7^–10^−5^ M ALN [42]. Thavornyutikarna et al. revealed enhanced TRAP activity of human THP-1-derived osteoclasts in the presence of 10^−6^–10^−9^ M ALN. Higher concentration was similar to the control group; lower concentration manifested an inhibition effect. Alternatively, a disruption of the actin ring was detected [35]. In another study, 10^−4^–10^−7^ M ALN was not found to inhibit bone resorption or TRAP-positive cells when rat osteoclasts were incubated on bone slices. On the other hand, in the same study, 10^−6^ M ALN inhibited osteoclast formation and resorption in bone marrow cell cultures [43]. Halasy-Nagy et al. reported the disruption of the formed actin ring of osteoclasts treated with 3 × 10^−5^ M ALN. Bone resorption was inhibited in the presence of 3 × 10^−5^ M ALN [7]. Inhibited bone resorption by osteoclasts was also observed when 5 × 10^−7^–5 × 10^−6^ M ALN was added to the culture media [44].

The positive ALN effect could be explained by the affirmative effect of BPs on cytokine release. Töyräs et al. detected an increased concentration of IL-6 from cultured RAW cells when treated with ALN [22]. IL-6 is a cytokine that positively affects the induction of osteoclastogenesis, independently of a RANK/RANKL signaling pathway. The mechanism of osteoclastogenesis induction is via an IL-6R receptor that binds IL-6; subsequently, the gp-130 signaling pathway is induced [45,46]. Differences obtained by many performed studies could be affected by different experimental settings, e.g., culture conditions, cell number, cultivation period, or cell type.

The future perspective of this study is to design a local drug delivery system that releases ALN at the site of the bone defect based on the tested concentrations of ALN. This system will help restore the properties of damaged bone tissue, favoring bone mass formation over bone resorption. The released ALN will act only for the desired period of time to renew the bone defect, thus omitting the risk of systemic delivery side effects.

## 5. Conclusions

In conclusion, a negative effect of the highest tested ALN concentration, 10^−6^ M ALN, was observed on the cultured rat and human PBMCs in in-vitro conditions. Surprisingly, a positive effect of low ALN concentrations, 10^−10^ and 10^−8^ M ALN, on the induction of osteoclastogenesis of PBMCs was detected. However, in the in vivo conditions, the situation is more complex. The interplay between different cell types, e.g., osteoclasts and osteoblasts, is orchestrated through the signaling pathways that are responsible for the release of cytokines and GFs that influence paracrine signaling. Therefore, we suggest concentrations of ALN between 10^−6^ and 10^−7^ M for further testing, as the concentration of 10^−7^ M was not cytotoxic and promoted the induction of osteoclastogenesis less than 10^−8^ and 10^−10^ M ALN.

## Data Availability

The data that support the findings of this study are available from the corresponding author upon reasonable request.

## Supplementary Materials

The following are available online at https://www.mdpi.com/2218-273X/11/3/438/s1. Figure S1: Rat peripheral blood mononuclear cells (rPBMCs) cultured in the absence of both M-CSF and RANKL (A); rPBMCs cultured in the presence of RANKL (B). The maximal value of detected DNA was approximately 12 ng/sample, as opposed to Figure 1A,B, with a maximum of approximately 40 ng/sample. Statistical significance is denoted above the columns. Statistical significance among the tested groups within one experimental day is given by sample name (black *p* < 0.05, red *p* < 0.001). rPBMCs staining using a tartrate-resistant acid phosphatase (TRAP) staining kit, Day 6, scale bar 100 µm, magnification 200×. Figure S2: Visualization of actin ring formation of hPBMCs on Day 15 using epifluorescence microscopy. Filamentous actin was stained with phalloidin (red color), and nuclei were stained with Hoechst 34580 (blue color); scale bar 100 µm, magnification 200×.
