# Supplementary figures and images for: The Effect of Alendronate on Osteoclastogenesis in Different Combinations of M-CSF and RANKL Growth Factors
Source: Biomolecules. 2021 Mar 16;11(3):438. doi: 10.3390/biom11030438 (PMC8035832; doi:10.3390/biom11030438)

**A** DNA quantification - rPBMCs

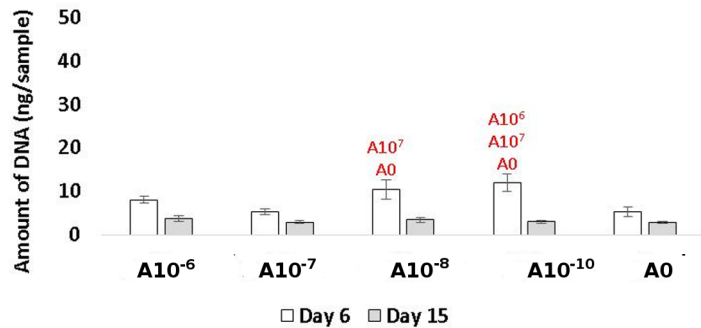

**B** DNA quantification - rPBMCs

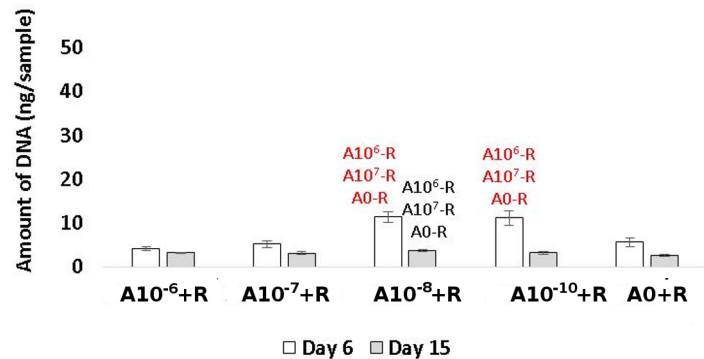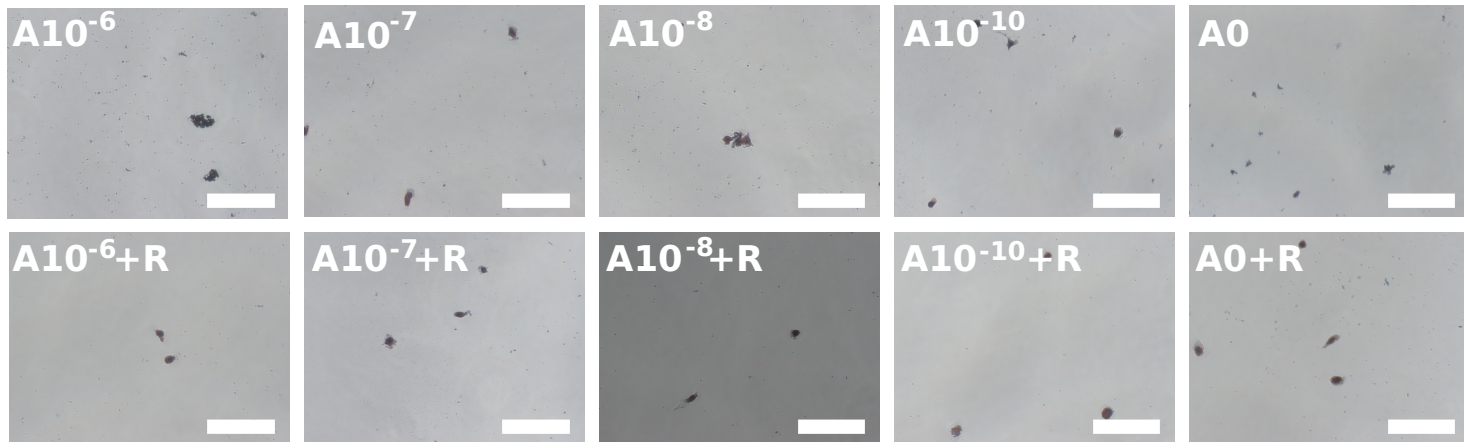

Supplement: Supplementary file 1 [file biomolecules-11-00438-s001.zip › Supplementary Figure S1.pdf]

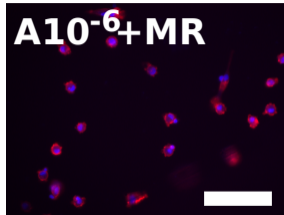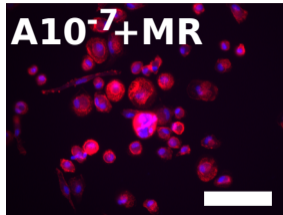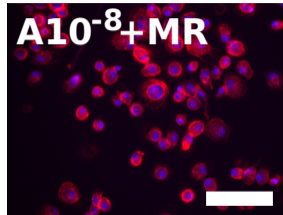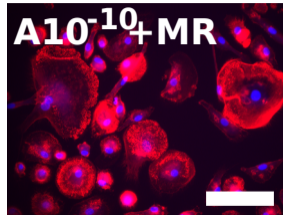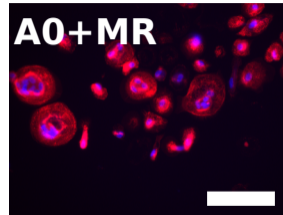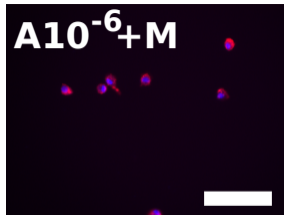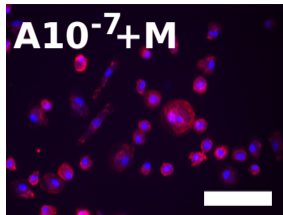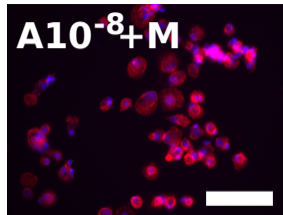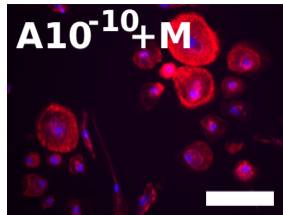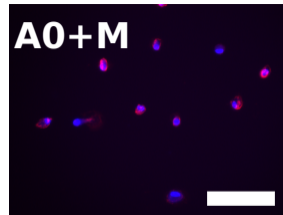

Supplement: Supplementary file 1 [file biomolecules-11-00438-s001.zip › Supplementary Figure S2.pdf]
